# Phase 2 Open-Label Study of Long-Term Safety, Tolerability, and Antiviral Activity of Rilpivirine in Antiretroviral-Naive Adolescents Living with HIV-1

**DOI:** 10.1128/aac.00916-21

**Published:** 2022-02-15

**Authors:** Johan Lombaard, Francis Ssali, Puthanakit Thanyawee, Jan Fourie, Simon Vanveggel, Cornelia Linthicum, Veerle Van Eygen, Rodica Van Solingen-Ristea

**Affiliations:** a Josha Research, Bloemfontein, South Africa; b Joint Clinical Research Centre, Kampala, Uganda; c Thai Red Cross AIDS Research Centre, Bangkok, Thailand; d Jan Fourie Medical Practice, Dundee, South Africa; e Janssen Research & Development, Beerse, Belgium; f Janssen Vaccines AG, Bern, Switzerland

**Keywords:** adolescents, antiretroviral therapy, efficacy, HIV-1, rilpivirine, safety

## Abstract

This phase 2 study investigated long-term safety and efficacy of rilpivirine (RPV) plus two investigator-selected nucleos(t)ide reverse transcriptase inhibitors (NRTIs) in HIV-1-infected antiviral therapy-naive adolescents. Participants (≥12 to <18 years of age) were treated with RPV at 25 mg once daily (q.d.) plus 2 NRTIs and entered the treatment extension period for up to 240 weeks, with visits every 3 months. Long-term safety (analysis of adverse events [AEs] and laboratory results), efficacy (virologic response and outcome for patients with viral loads of <50 and <400 by time to loss of virologic response [TLOVR] and FDA Snapshot methods, as well as CD4^+^ cell count), and adherence (by pill count) for up to 240 weeks are presented. Twenty-four of 36 participants entered the treatment extension period, and 21 completed week 240. At week 240, a viral load of <50 copies/mL was achieved by 14/32 (43.8%) participants; virologic response by TLOVR was higher in participants with a baseline viral load of ≤100,000 copies/mL (48.0%) versus a viral load of >100,000 copies/mL (28.6%). By FDA Snapshot, a viral load of <50 copies/mL at week 240 was found in 53.1% (17/32) of participants with a baseline viral load of ≤100,000 copies/mL. Higher response was observed in participants with adherence of >95% and a baseline viral load of ≤100,000 copies/mL. Through week 240, 16/32 participants (50.0%) experienced virologic failure, including seven who developed treatment-emergent RPV resistance-associated mutations (RAMs [frequently E138K]): all 7 had ≥1 treatment-emergent NRTI RAM. No serious AEs after week 48, no discontinuations due to AEs between week 48 and week 240, and no new safety signals were observed. RPV did not affect pubertal development or adolescent growth. At the 5-year follow-up, efficacy was low in adolescents, particularly those with poor adherence and/or a high baseline viral load of >100,000 copies/mL. To limit the risk of virologic failure, RPV is restricted to patients with a baseline VL of ≤100,000 copies/mL in most countries. In addition, adequate treatment adherence to RPV treatment is imperative for long-term viral suppression and should be emphasized in the management of adolescents living with HIV. RPV exhibited a favorable long-term safety profile for adolescents living with HIV-1 with adequate adherence. (This study has been registered at ClinicalTrials.gov under identifier NCT00799864.)

## INTRODUCTION

Long-term data on treatment of adolescents living with HIV-1 are limited (1, 2). Inadequate treatment adherence to antiretroviral therapy (ART) is a major challenge among adolescents living with HIV (3), which leads to reduced treatment response, potentially impacting future treatment options (4, 5).

Rilpivirine (RPV [Edurant]), a nonnucleoside reverse transcriptase inhibitor (NNRTI), was approved (25-mg once-daily [q.d.] tablet) for treatment of HIV-1 treatment-naive adults based on the results of the phase 3 ECHO and THRIVE studies. RPV at 25 mg q.d. demonstrated noninferior efficacy compared with efavirenz (EFV) at 600 mg q.d., both in combination with two nucleoside/nucleotide RTIs (NRTIs) (6–8).

The PAINT study (ClinicalTrials registration no. NCT00799864) is a phase 2, open-label study of RPV in HIV-1-infected, ART-naive adolescents ≥12 to <18 years of age (cohort 1) and children ≥6 to <12 years of age (cohort 2). The 48-week analysis of cohort 1 of this study established that the pharmacokinetic (PK), safety, and resistance profile of RPV (25 mg q.d.) in adolescents was comparable to that in adults (9). The 48-week analysis also supported regulatory approval for use of RPV at 25 mg q.d. plus 2 NRTIs in HIV-1-infected treatment-naive adolescents with an HIV-1 plasma viral load (VL) of ≤100,000 copies/mL. The 24- and 48-week results were previously published (9, 10). Here, we report the final analysis for cohort 1, including the long-term efficacy, safety, and tolerability of RPV for up to 240 weeks of treatment, in combination with 2 investigator-selected NRTIs, in HIV-1-infected, ART-naive adolescents.

## RESULTS

### Patient disposition and baseline characteristics.

**FIG 1:**
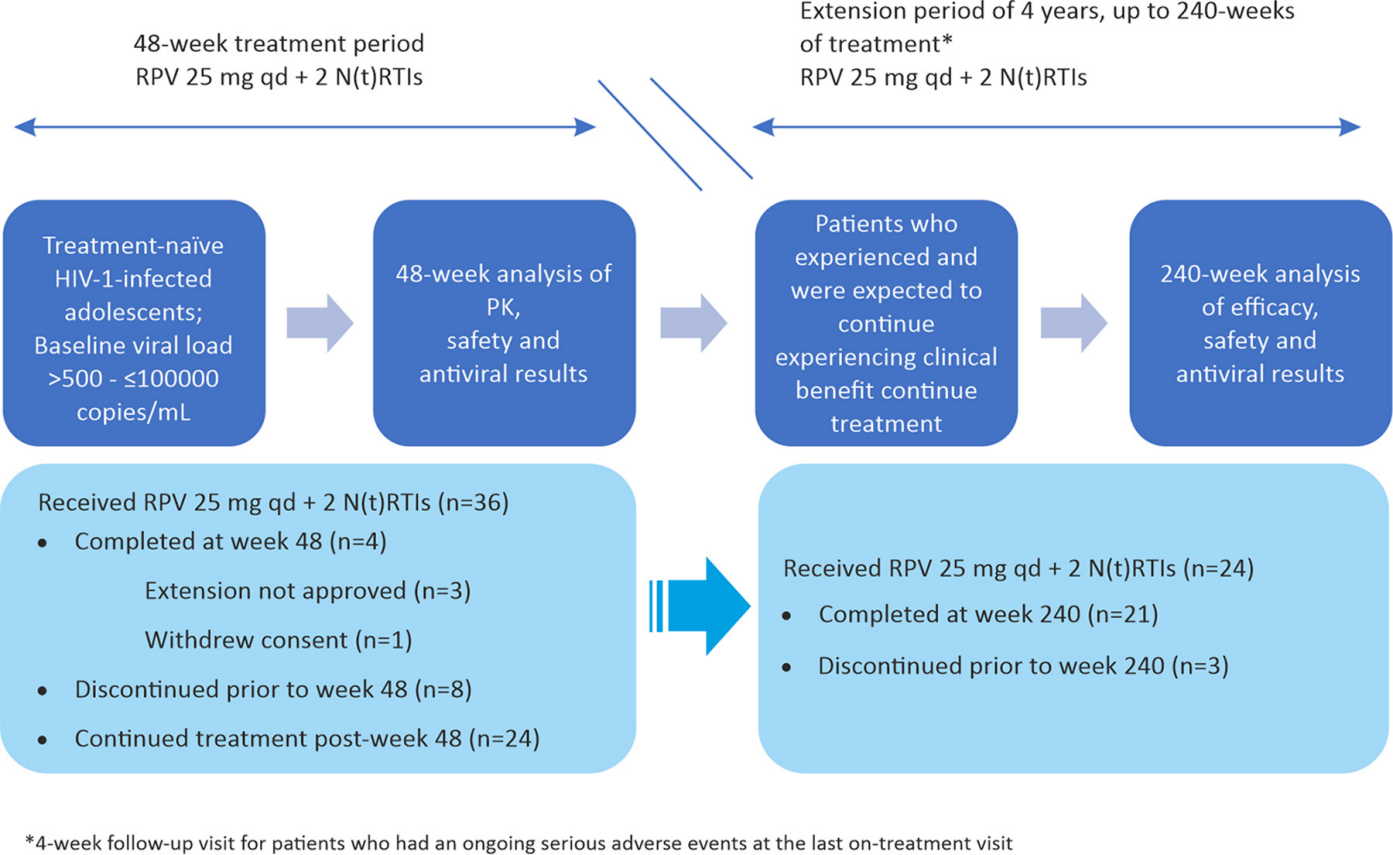
Study design. HIV, human immunodeficiency virus; N(t)RTIs, nucleos(t)ide analogue reverse transcriptase inhibitor; PK, pharmacokinetics; q.d., once daily; RPV, rilpivirine.

The study was conducted at 7 sites across South Africa, India, Thailand, the United States, and Uganda, and 4/7 sites (2 sites in South Africa and 1 each in Thailand and Uganda) participated in the post-week 48 treatment extension period. An overall summary of the study design is presented in Fig. 1. Of 71 screened patients, 36 were enrolled and treated. One patient withdrew consent, and for 3 patients, the local health authority approval for post-week 48 follow-up was not obtained for reasons not related to participation; these 4 patients were not included in the intent-to-treat (ITT) population for efficacy analyses after week 48. Of the 32 patients included in the post-week 48 efficacy analyses, 8 discontinued (1 for an adverse event [AE], 6 for reaching a virologic endpoint, and 1 for unspecified reasons), all prior to week 48. Of 24 patients (66.7%) entering the extension period, 21 (58.3%) completed study at week 240; 3 (8.3%) discontinued before week 240 as they reached a virologic endpoint. At baseline, 28 patients (77.8%) had a VL of ≤100,000 copies/mL, and 8 patients (22.2%) had a VL of >100,000 copies/mL. Major protocol deviations were observed in 6 patients through week 240 (Table 1). The majority of patients (24/36 [66.7%]) received a combination of emtricitabine (FTC) plus tenofovir disoproxil fumarate (TDF) as the background NRTI regimen. The other NRTIs used were lamivudine (3TC) plus TDF (8 [22.2%]) and 3TC plus zidovudine (4 [11.1%]). The total number of patient-years of exposure was 109.7.

**TABLE 1:** Patient baseline demographics and disease characteristics for the intent-to-treat population^*a*^

Characteristic	RPV at 25 mg q.d. (*n* = 36)
Sex, *n* (%)	
Female	20 (55.6)
Male	16 (44.4)
Age (yr), mean (SD)	14.6 (1.66)
≥12 to <15 yr, *n* (%)	18 (50.0)
≥15 to <18 yr, *n* (%)	18 (50.0)
Race, *n* (%)	
Asian	4 (11.1)
Black or African-American	32 (88.9)
Ethnicity, *n* (%)	
Not Hispanic or Latino	36 (100.0)
Body mass index, kg/m², mean (SD)	19.56 (3.73)
HIV-1 clade, *n* (%)	
A1	9 (25.0)
B	1 (2.8)
C	23 (63.9)
CRF01_AE	1 (2.8)
D	2 (5.6)
Hepatitis B/C coinfection status, *n* (%)	3 (8.3)
Baseline HIV-1 RNA	
Copies/mL, mean (SD)	115,942.5 (169,149.69)
Categorical, *n* (%)	
≤100,000 copies/mL	28 (77.8)
>100,000 to ≤500,000 copies/mL	6 (16.7)
>500,000 copies/mL	2 (5.6)
Baseline CD4^+^ cell count	
Cells/μL, mean (SD)	426.6 (215.11)
%, mean (SD)	20.75 (10.053)
Categorical, *n* (%)	
≤200 cells/μL	4 (11.1)
>200 cells/μL	32 (88.9)
Mode of HIV infection, *n* (%)	
Heterosexual contact	4 (11.1)
Mother-to-child transmission	30 (83.3)
Other	1 (2.8)
Unknown	1 (2.8)
Any major protocol deviation, *n* (%)	6 (16.7)
Received wrong treatment or incorrect dose	4 (11.1)
Entered but did not satisfy criteria	1 (2.8)
Received an excluded concomitant treatment	1 (2.8)
Other^*b*^	1 (2.8)

a HIV, human immunodeficiency virus, q.d., once daily; RPV, rilpivirine; SD, standard deviation.

b One participant had two protocol deviations: food requirement adherence and received wrong treatment or incorrect dose.

### Efficacy. (i) Virologic response.

**FIG 2:**
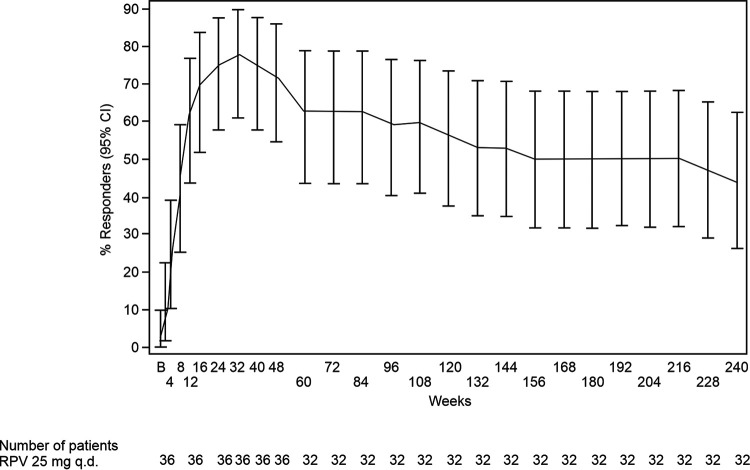
Virologic response (<50 copies/mL) by the time to loss of virologic response (TLOVR) approach over time; intent to treat population. B, baseline; CI, confidence interval; q.d., once daily; RPV, rilpivirine.

Per the TLOVR (time to loss of virologic response [VR]) algorithm, VR (<50 copies/mL) was observed in 26/36 (72.2%) patients at week 48 and 14/32 patients (43.8%) at week 240 (Fig. 2). Applying 400 copies/mL as the threshold, VR was observed in 27/36 patients (75.0%), and 16/32 patients (50.0%) at weeks 48 and 240, respectively. The VR (<50 copies/mL) rate using FDA Snapshot at week 240 was 17/32 patients (53.1%), as 3 patients with confirmed virologic rebound who were considered to show virologic failure (VF) by TLOVR resuppressed (confirmed) at week 240. At week 240, the percentage of patients with VR was higher in patients with a baseline VL of ≤100,000 copies/mL (12/25 [48.0%] by TLOVR and 14/25, 56% by FDA Snapshot) versus a VL of >100,000 copies/mL (2/7 [28.6%] by TLOVR and 3/7 [42.9%] by Snapshot) (Table 2). VR was observed in 12/25 patients (48.0%) in a subgroup with treatment adherence of >95% versus <95% (2/7 patients [28.6%]). The VR rate at week 240 in the subgroup with a baseline VL of ≤100,000 copies/mL and treatment adherence of >95% was 11/21 patients (52.4%), compared with 3/11 patients (27.3%) in the subgroup with a baseline plasma VL of >100,000 copies/mL or treatment adherence of ≤95% (see Table S1 in the supplemental material). There were 9 (50%) patients with VR 12 to <15 years of age and 5 (35.7%) among those ≥15 years of age (see Table S2 in the supplemental material).

**TABLE 2:** Virologic outcome at week 240 by TLOVR and FDA Snapshot analysis^*a*^

Outcome	Outcome, *n* (%)		
	RPV at 25 mg q.d. (*n* = 32)	BL subgroup	
		VL of ≤100,000 copies/mL (*n* = 25)	VL of >100,000 copies/mL (*n* = 7)
TLOVR		
Responders (VL of <50 copies/mL)	14 (43.8)	12 (48.0)	2 (28.6)
Virologic failure	16 (50.0)	12 (48.0)	4 (57.1)
Never suppressed	4 (12.5)	2 (8.0)	2 (28.6)
Initial lack of response	1 (3.1)	0	1 (14.3)
Rebounder	12 (37.5)	10 (40.0)	2 (28.6)
Resuppressed (confirmed) afterwards	3 (9.4)	2 (8.0)	1 (14.3)
Discontinued due to AE	1 (3.1)	0	1 (14.3)
Discontinued due to reason other than AE	1 (3.1)	1 (4.0)	0

Snapshot analysis^*b*^			
Virologic success	17 (53.1)	14 (56.0)	3 (42.9)
HIV-1 RNA of <50 copies/mL at wk 240	17 (53.1)	14 (56.0)	3 (42.9)
Virologic failure	14 (43.8)	11 (44.0)	3 (42.9)
HIV-1 RNA of ≥50 copies/mL at wk 240	4 (12.5)	4 (16.0)	0
Virologic failure leading to discontinuation	9 (28.1)	6 (24.0)	3 (42.9)
Virologic failure—discontinued due to other reason and last available HIV-1 RNA of ≥50 copies/mL	1 (3.1)	1 (4.0)	0
No VL data in 240-wk window	1 (3.1)	0	1 (14.3)
Discontinued due to AE/death	1 (3.1)	0	1 (14.3)

a TLOVR, time to loss of virologic response; AE, adverse events; BL, baseline; HIV, human immunodeficiency virus; q.d., once daily; VL, viral load; RPV, rilpivirine.

b FDA Snapshot analysis results are presented for the intent-to-treat population.

### (ii) Virologic failure.

At the week 240 analysis, 16/32 patients (50.0%) were considered VF (TLOVR), of whom 4 were never suppressed and 12 were rebounders (2 consecutive visits with ≥50 copies/mL, with 3/12 patients subsequently resuppressed) (Table 2); 2/32 patients (6.3%) discontinued prior to week 240 for reasons other than reaching a virologic endpoint.

VF was observed in 12/25 patients (48.0%) with treatment adherence of >95% versus 4/7 patients (57.1%) with <95% treatment adherence. The VF rate with a baseline VL of ≤100,000 copies/mL and treatment adherence of >95% was 9/21 (42.9%), versus 7/11 (63.6%) with a baseline VL of >100,000 copies/mL and treatment adherence of ≤95% (Table S1).

Stratified by age group, VF rates in older adolescents (15 to <18 years old) versus younger adolescents (12 to <15 years old) were 8/14 (57.1%) and 8/18 (44.4%), respectively (Table S2).

### (iii) Change in CD4^+^ cell count.

The mean (standard error [SE]) increases from baseline in absolute CD4 count were 201.2 (32.87) cells/μL at week 48 and 113.6 (26.72) cells/μL at week 240 based on the “noncompleter = failure” (NC = F) approach, with 255.1 (36.12) cells/μL at week 48 and 178.0 (37.67) cells/μL at week 240 based on observed case analysis.

### Adherence.

Mean RPV treatment adherence (based on pill count) through week 240 was 97.52%. Most patients had >95% adherence (28/36 [77.8%]), and 8/36 (22.2%) patients had ≤95% adherence. Overall, treatment adherence was ≥80% in all the patients. However, considerable within-patient fluctuation was observed throughout the study: 7 patients (21.9%) had ≥1 24-week episode with <80% adherence. However, no clear association with VF (TLOVR) was observed. Of note, some patients had >100% compliance at all scheduled visits, which was indicative of poor adherence (11; data not shown).

### Antiretroviral resistance.

Among 15 VFs with post-baseline genotypic data, 8/32 (25.0%) patients did not carry treatment-emergent RPV resistance-associated mutations (RAMs), while 7/32 (21.9%) had ≥1 treatment-emergent RPV RAM at the last post-baseline time point with genotypic data in the week 240 analysis compared to 5/36 patients (13.9%) in the week 48 analysis. Three of seven patients with phenotypic data showed reduced susceptibility to RPV at the last post-baseline time point with genotypic data.

The most frequent treatment-emergent RPV RAM at the last post-baseline time point was E138K (5/15 patients [33.3%]). All 7 patients with RPV RAMs also had at least 1 treatment-emergent NRTI RAM at the last post-baseline time point with genotypic data. The most frequent treatment-emergent NRTI RAM was M184V (4/15 patients [26.7%]). In addition, K65R plus Y115F (*n* = 1) and M184I (*n* = 2) were observed in combination with RPV RAMs. Three patients had both treatment-emergent RPV RAM E138K and NRTI RAM M184V at the last post-baseline time point with genotypic data.

### Safety.

At week 240, the mean (SD) increases from baseline in height, weight, and body mass index (BMI) were 10.2 (8.04) cm, 15.7 (7.14) kg, and 3.8 (3.58) kg/m^2^, respectively. During the overall treatment phase, adverse events (AEs) were reported in 35/36 patients (97.2%); such events were reported in 22/24 patients (91.7%) after week 48 (Table 3). The most frequently reported AEs (>20% patients) were upper respiratory tract infection (55.6%), influenza (41.7%), cough (27.8%), headache (25.0%), and depression (22.2%). No serious AEs (SAEs) were reported, and no patients discontinued the study due to AEs after week 48. AEs considered possibly related to RPV by investigator were reported in 1 patient (4.2%) after week 48 (headache). Grade 3 or 4 AEs were reported in 7 patients (19.4% [5 patients before week 48 and 2 patients after week 48]); no grade 3 or 4 AE was reported as at least possibly related to RPV.

**TABLE 3:** Incidence of adverse events from the intent-to-treat analysis set

AE(s)^*a*^	*n* (%) with RPV at 25 mg q.d.		
	1st 48 wk (*n* = 36)	wk 48–240 (*n* = 24)	Overall treatment phase (*n* = 36)
Patients with ≥1 AE	34 (99.4)	22 (91.7)	35 (97.2)

AEs occurring in >10% of patients			
Upper respiratory tract infection	8 (22.2)	15 (62.5)	20 (55.6)
Blood cortisol decrease	7 (19.4)	0	7 (19.4)
wt decrease	4 (11.1)	0	4 (11.1)
Headache	5 (13.9)	5 (20.8)	9 (25.0)
Somnolence	5 (13.9)	0	5 (13.9)
Bronchitis	4 (11.1)	0	4 (11.1)
Influenza	13 (36.1)	4 (16.7)	15 (41.7)
Urinary tract infection	2 (5.6)	3 (12.5)	4 (11.1)
Dental caries	2 (5.6)	3 (12.5)	3 (8.3)
Cough	8 (22.2)	3 (12.5)	10 (27.8)
Nausea	4 (11.1)	1 (4.2)	5 (13.9)
Depression	7 (19.4)	2 (8.3)	8 (22.2)

Patients with ≥1 SAE	6 (16.7)	0	6 (16.7)
AEs leading to study discontinuations	1 (2.8)	0	1 (2.8)
AEs leading to deaths	0	0	0
Patients with ≥1 AE related to RPV	13 (36.1)	1 (4.2)	13 (36.1)

AEs with ≥1 DAIDS grade 3/4	5 (13.9)	2 (8.3)	7 (19.4)
Depression	1 (2.8)	1 (4.2)	2 (5.6)
Neutropenia	0	1 (4.2)	1 (2.8)
Pancreatitis	1 (2.8)	0	1 (2.8)
Blood phosphorus decreased	1 (2.8)	0	1 (2.8)
Malaria	2 (5.6)	0	2 (5.6)
Abscess limb	1 (2.8)	0	1 (2.8)
Suicidal ideation	1 (2.8)	0	1 (2.8)
Suicide attempt	1 (2.8)	0	1 (2.8)

AEs of special interest	24 (66.7)	8 (33.3)	26 (72.2)
AEs of special interest in >10% of patients			
Headache	5 (13.9)	5 (20.8)	9 (25.0)
Somnolence	5 (13.9)	0	5 (13.9)
Dizziness	3 (8.3)	2 (8.3)	4 (11.1)
Depression	7 (19.4)	2 (8.3)	8 (22.2)
Blood cortisol decreased	7 (19.4)	0	7 (19.4)
Rash	4 (11.1)	0	4 (11.1)

a AE, adverse event; DAIDS, Division of AIDS; q.d., once-daily; RPV, rilpivirine; SAE, serious adverse event.

AEs of special interest were reported in 26/36 patients (72.2%) during the overall treatment phase: 24/36 patients (66.7%) before week 48 and 8/24 patients (33.3%) after week 48. The most frequently reported individual AEs of special interest after week 48 were headache, depression, and dizziness. Increases in QTcB and QTcF of >60 ms from baseline were observed in 1 patient (2.8%) at week 240 and resulted in unconfirmed pathologically prolonged (>500-ms) QTcB and QTcF values (591 and 520 ms, respectively), which were not reported as AEs. There were no pregnancies reported during the study.

Most laboratory abnormalities were grade 1 or grade 2. Between week 48 and week 240, no grade 4 laboratory abnormalities were observed; grade 3 abnormalities in neutrophils and precursors, neutrophils segmented, and hemoglobin were reported in 1 patient each. Mean (SD) increases from baseline in creatinine were 6.6 (7.35) μmol/L at week 48 and 14.3 (12.12) μmol/L at week 240. Although a mean increase from baseline creatinine of 14.3 μmol/L (0.16 mg/mL) was observed at week 240, in only 3 patients was a decrease from baseline glomerular filtration rate (GFR) of more than 30% observed at any time. The 3 patients had a high renal function (>120 mL/min/1.73 m^2^) at baseline and had a normal or high renal function (90 to 120 mL/min/1.73 m^2^) at the time of the largest decrease from baseline in GFR. All of these 3 patients used TDF as a concomitant antiretroviral (ARV).

There were no consistent mean changes from baseline in basal cortisol levels over the overall treatment phase: mean (SD) basal cortisol levels were 229.3 (73.57) nmol/L at baseline, 261.0 (114.42) nmol/L at week 48, and 237.8 (58.08) nmol/L at week 240. Adrenocorticotropic hormone (ACTH) stimulation test results were normal (cortisol, ≥500 nmol/L after stimulation) in most patients at week 48 (22/29 patients [75.9%]), at week 240 (8/9 patients [88.9%]), and at the last measurement available on treatment (34/36 patients [94.4%]). RPV did not affect pubertal development (assessed by Tanner staging) or growth of adolescents (assessed by CDC growth curves). No AEs related to clinical manifestations of adrenal insufficiency were reported.

## DISCUSSION

The long-term efficacy of RPV at 25 mg q.d. in combination with 2 investigator-selected NRTIs in HIV-1 in ART-naive adolescents living with HIV-1 (≥12 to <18 years of age) was evaluated in this 240-week, phase 2 study (PAINT). VR (<50 copies/mL) was achieved in 72.2% of patients at week 48 and 43.8% of patients at week 240 (TLOVR approach). In patients with a baseline VL of ≤ 100,000 copies/mL, VR was achieved in 12/25 patients (48.0%) at week 240 by TLOVR and in 14/25 patients (56%) by FDA Snapshot. At week 48, this VR rate was lower in this study (73%) than in adult RPV studies at week 48 (6). The same differences have been observed when comparing published longer-term data from adolescents and adults. Indeed, recent evidence showed that >50% of adolescents do not maintain virological suppression despite prolonged ARV therapy (12). This difference was also observed in a retrospective real-world study, wherein adolescents and young adults had lower rates of HIV-1 virologic suppression and higher rates of HIV-1 viral rebound and failure to follow-up compared to adults (13). Adolescents in general have higher risk of virological failure than adults (14).

In the PAINT study, VR at week 240 was observed in 12/25 (48.0%) of patients with adherence of >95% and in 2/7 (28.6%) patients with adherence of ≤95%. Of note, VR was higher in the subgroup with a baseline plasma viral load of ≤100,000 copies/mL combined with treatment adherence of >95% (11/21 patients [52.4%]), compared to 3/11 patients (27.3%) in the subgroup with a baseline VL of >100,000 copies/mL or treatment adherence of ≤95%. There is limited long-term data on the efficacy of ARVs in treatment-naive adolescents. In a 96-week study, a once-daily regimen of emtricitabine, didanosine, and efavirenz, achieved a VL of <50 copies/mL in 72% of therapy-naive adolescents and children living with HIV-1 who were 3 to 21 years of age (15). Results from up to 144 weeks of the IMPAACT P1093 study suggest that dolutegravir along with a background regimen was safe and efficacious in HIV-1-infected, treatment-experienced adolescents 12 to <18 years of age, with an HIV-1 RNA level ≥1,000 copies/mL at entry in the study. Longer-term (144 weeks) data from the P1093 study of dolutegravir in treatment-experienced adolescents showed in the ITT analysis, virologic success (VL of <400 copies/mL at week 144) was achieved in 10 of 23 participants (43%; 95% confidence interval, 23.2 to 65.5%), and 8 of 23 (35%; 16.4 to 57.3%) had an HIV-1 RNA level of <50 copies/mL at week 144 (16). However, most who experienced virologic failure had adherence levels of <90%, suggesting adherence-related challenges are common in this age group of adolescents (16). Although the study population of the P1093 study is treatment-experienced adolescents, while study C213 has a treatment-naive adolescent population, these results emphasize the challenges in achieving long-term efficacy in adolescents with HIV. In studies with other ARVs and longer follow-ups, VRs (VL of <50 copies/mL) varied from 70% at 192 weeks (75/107 patients <15 years of age [treatment naive]) (17) to 2% at week 292 (1/53 patients 12 to 18 years [not treatment naive]) (18).

It must be acknowledged that pill count is a suboptimal instrument to measure adherence as actual pill intake was not directly observed. Furthermore, other critical factors, such as adherence to RPV intake with food, could have played a vital role. Hence, these limitations must be considered while interpreting the results.

The WHO recommends drug optimization for children and adolescents with HIV, phasing out NNRTI-based regimens and replacing them with optimal regimens and formulations as outlined in the 2018 Optimal Formulary and Limited-Use List for Pediatric ARVs (19). In those countries where transition to dolutegravir is promoted, a first-line dolutegravir-based regimen should be provided to all adolescents weighing more than 25 kg (tenofovir, lamivudine, and dolutegravir [TLD] for those more than 30 kg), in line with national guidance on dolutegravir use in adolescent girls of childbearing potential (20).

No safety concerns were noted in this long-term study with RPV in HIV-1-infected, ART-naive adolescents. The data from adolescents confirm the overall favorable safety profile of RPV, consistent with the established safety profile in adults, with no deaths overall, no SAEs, and no AEs leading to treatment discontinuation after week 48 through week 240. During the overall treatment phase, 35 patients (97.2%) experienced ≥1 AE: the most frequent were upper respiratory tract infection (20/32 [55.6%]), influenza (15/32 [41.7%]), cough (10/32 [27.8%]), headache (9/32 [25.0%]), and depression (8/32 [22.2%]). Most AEs (including grade 3 to 4 AEs, AEs related to RPV or the underlying ART, and HIV-related AEs) emerged in the first 48 weeks of treatment, and no new safety concerns were observed thereafter.

In the PAINT study, the ACTH stimulation results were normal (cortisol levels of ≥500 nmol/L) over 240 weeks of RPV treatment in the majority of patients during the overall treatment phase. Also, pubertal development, as assessed by Tanner staging (4, 21) and growth of adolescents, was not affected by RPV treatment in this long-term study.

The observed RPV resistance data were in line with the phase 3 ECHO and THRIVE studies (22, 23). The most common treatment-emergent RPV and NRTI RAMs were E138K and M184V, respectively.

### Conclusion.

In summary, week 240 results from cohort 1 (adolescents) in the PAINT phase 2 study showed that when RPV at 25 mg q.d. was used in combination with 2 investigator-selected NRTIs in treatment-naive adolescents with a baseline VL of ≤100,000 copies/ml, 56% of patients were responders by FDA Snapshot (16/32 participants [50.0%] had virologic failure). Patients with VF and potentially developing RAMs had a tendency to show poor adherence, along with a higher baseline VL. No new safety concerns were observed in this patient population, aside from the known safety profile of RPV in adults. Moreover, pubertal development and growth of adolescents were not affected by long-term treatment with RPV. Study limitations include small sample size, the open-label and nonrandomized design, the absence of a comparator arm, and challenges in accurate measurement of adherence.

To limit the risk of virologic failure, in most countries RPV is restricted to patients with a baseline VL of ≤100,000 copies/mL. In addition, adequate treatment adherence is imperative for long-term viral suppression and should be emphasized in the management of adolescents living with HIV.

## MATERIALS AND METHODS

### Study design.

PAINT (ClinicalTrials.gov no. NCT00799864; TMC278-TiDP38-C213) is a phase 2, single-arm, open-label, multicenter study of RPV in ART-naive adolescents living with HIV-1 who are ≥12 to <18 years of age (cohort 1) and children ≥6 to <12 years of age (cohort 2). The study consists of a ≤8-week screening period and a 48-week initial treatment period (48 weeks), followed by a 4-year treatment extension period (up to 240 weeks) for participants who are expected to continue experiencing clinical benefit from RPV plus 2 NRTIs at the end of 48 weeks (Fig. 1). For cohort 1, the study was conducted between December 2010 and April 2018. The study was completed for subjects in cohort 1 but is ongoing for cohort 2. This article describes the long-term results of the week 240 final analysis for cohort 1.

This study was conducted in compliance with the Declaration of Helsinki and was consistent with Good Clinical Practices and applicable regulatory requirements. The study protocol and amendments were reviewed by the independent ethics committees or institutional review boards. Written informed consent was obtained from all participants and/or legally acceptable representatives before study enrollment.

### Participants.

Adolescents (boys or girls ≥12 to <18 years of age with a body weight of ≥32 kg) with documented HIV-1 infection and who had never been treated with an HIV vaccine/drug were enrolled. Following approval in 2011 of RPV in adults with a viral load of ≤100,000 HIV-1 RNA copies/mL, enrollment in cohort 1 was restricted to screening HIV-1 plasma VLs of ≤100,000 copies/mL. The patients’ VL at screening had to be between ≥500 and ≤100,000 HIV-1 RNA copies/mL. Patients with previously documented HIV-2 infection, with active AIDS, and with documented genotypic evidence of ≥1 NNRTI resistance-associated mutation (RAM) from a predefined list of the following NNRTI RAMs (24, 25) at screening were excluded: A98G, L100I, K101E, K101P, K101Q, K103H, K103N, K103S, K103T, V106A, V106M, V108I, E138A, E138G, E138K, E138Q, E138R, V179D, V179E, V179T, Y181C, Y181I, Y181V, Y188C, G190A, G190C, G190E, G190Q, G190S, G190T, P225H, F227C, Y188H, Y188L, M230I, M230L, P236L, K238N, K238T, and Y318F.

### Treatment regimens.

All patients received RPV 25 mg q.d. plus an investigator-selected background regimen containing 2 NRTIs (restricted to zidovudine [AZT], abacavir [ABC], or tenofovir disoproxil fumarate [TDF] in combination with either lamivudine [3TC] or emtricitabine [FTC]) in an age-appropriate formulation based on marketing approval or per the local standard of care.

### Efficacy assessments.

The primary efficacy endpoint represented the proportion of patients with confirmed and sustained virologic response (VR [VL of <50 copies/mL]) according to the time to loss of VR (TLOVR) algorithm at week 24 and was presented in a previous publication (10). The secondary efficacy endpoints at week 240 were the proportion of patients with VR using the FDA Snapshot approach (26), TLOVR (27), and change in CD4^+^ cell count (absolute and percentage) from baseline up to week 240.

Plasma VL was assessed at prespecified time points using the Roche Cobas Amplicor HIV-1 Monitor test, version 1.5 (lower limit of quantification [LLOQ], 50 copies/mL [until the end of 2012]), or the Roche Cobas TaqMan HIV-1 test, version 2.0 (LLOQ, 20 copies/mL [after 2012]).

### Virology assessments.

Resistance determination was performed at screening, baseline, early withdrawal, and at virologic failure (VF). Viral genotyping and phenotyping were conducted using VircoTYPE and Antivirogram assays or the PhenoSense GT assay. The RPV fold change (FC) in 50% effective concentration values was assessed using a RPV biological cutoff (BCO) of 3.7 in Antivirogram and 2.0 in PhenoSense GT (22, 28).

### Safety assessments.

Safety assessments included adverse events (AEs), serious AEs (SAEs), AEs related to study drug, AEs leading to discontinuations, AEs of interest (skin, neuropsychiatric, potential QT prolongation related, hepatic, endocrinology, and AIDS-defining illnesses), and grade 3 to 4 AEs at week 240.

Laboratory data were collected for hematology, biochemistry, endocrine assessments, and urinalysis. The basal cortisol was measured in the morning, between 7:30 and 9:30 a.m. Vital and physical signs and electrocardiograms were recorded at predefined time points. Growth and development parameters were measured every 6 months during the treatment extension period. AEs were coded in accordance with Medical Dictionary for Regulatory Activities (MedDRA), version 13.1. The Division of AIDS (DAIDS) severity grading list (December 2004) was used to grade AEs and laboratory abnormalities.

### Adherence evaluation.

Adherence questionnaires were used to assess adherence during the initial 48-week treatment period, and the results were described in the week 48 analysis (9). After the week 48 visit, adherence was assessed based on pill count collected in the electronic case report form. Drug accountability by pill count was performed at each visit throughout the study (at weeks 2, 4, 8, 12, and 16, every 8 weeks to week 48, and then every 3 months to week 240). The site staff counted the medication brought by the participant to the site for pill count.

### Statistical analysis.

The efficacy and safety analyses were performed on the intent-to-treat (ITT) population, defined as the set of all patients who received at least 1 dose of RPV, regardless of their compliance with protocol and adherence to dosing regimen. The ITT population for efficacy analyses consisted of 36 patients prior to week 48 and 32 patients after week 48 (excluding 4 who completed the study at week 48). Change from baseline in CD4^+^ cell count was performed on imputed data using the approach “noncompleter = failure” (NC=F)—i.e., imputed by baseline value after discontinuation (change = 0)—or last observation carried forward (LOCF) otherwise, as well as the observed case. The ITT population for safety analyses consisted of 36 patients for first 48 weeks of treatment, 24 patients between week 48 and week 240, and 36 patients for the overall 240-week treatment phase.
