# Supplementary material for: Phase 2 Open-Label Study of Long-Term Safety, Tolerability, and Antiviral Activity of Rilpivirine in Antiretroviral-Naive Adolescents Living with HIV-1
Source: Antimicrob Agents Chemother. 2022 Feb 15;66(2):e00916-21. doi: 10.1128/aac.00916-21 (PMC8846324; doi:10.1128/aac.00916-21)
Supplement: Supplemental file 1 — Supplemental material. Download aac.00916-21-s0001.pdf, PDF file, 0.3 MB [file aac.00916-21-s0001.pdf]

Supplementary Table 1: Virologic Outcome (TLOVR) at Week 240 by Baseline Viral Load and Adherence; Intent to treat

	RPV 25 mg q.d. (N=32)	
	**VL ≤100000 copies/mL and adherence >95% (N=21)**	**VL >100000 copies/mL OR adherence ≤95% (N=11)**
Responder	11 (52.4%)	3 (27.3%)
Virologic failure	9 (42.9%)	7 (63.6%)
never suppressed	1 (4.8%)	3 (27.3%)
initial lack of response	0	1 (9.1%)
rebounder	8 (38.1%)	4 (36.4%)
re-suppressed (confirmed) afterwards	2 (9.5%)	1 (9.1%)
Discontinued due to AE	0	1 (9.1%)
Discontinued due to reason other than AE	1 (4.8%)	0

Imputation method: TLOVR (<50 copies/mL)

AE: adverse events; N: number of patients with data, n: number of patients with that observation; RPV: rilpivirine; TLOVR: Time to Loss of Virologic Response; VL: viral load

Supplementary Table 2: Virologic Outcome at Week 240 by Age Category at Baseline by Time to Loss of Virologic Response (TLOVR)

	Age Category at Baseline	
**Outcome, n (%)**	**≥12 - <15 Years (N=18)**	**≥15 - <18 Years (N=14)**
Responders (VL <50 copies/mL)	9 (50.0)	5 (35.7)
Virologic failure	8 (44.4)	8 (57.1)
Never suppressed	2 (11.1)	2 (14.3)
Initial lack of response	0	1 (7.1)
Rebounder	6 (33.3)	6 (42.9)
Resuppressed (confirmed) afterwards	2 (11.1)	1 (7.1)
Discontinued due to AE	1 (5.6)	0
Discontinued due to reason other than AE	0	1 (7.1)

AE: adverse events; VL: viral load; TLOVR: Time to Loss of Virologic Response

Supplementary Figure 1: Mean (±SE) Changes From Baseline in CD4+ Cell Counts (cells/µL) Over Time; Intent-to-treat population

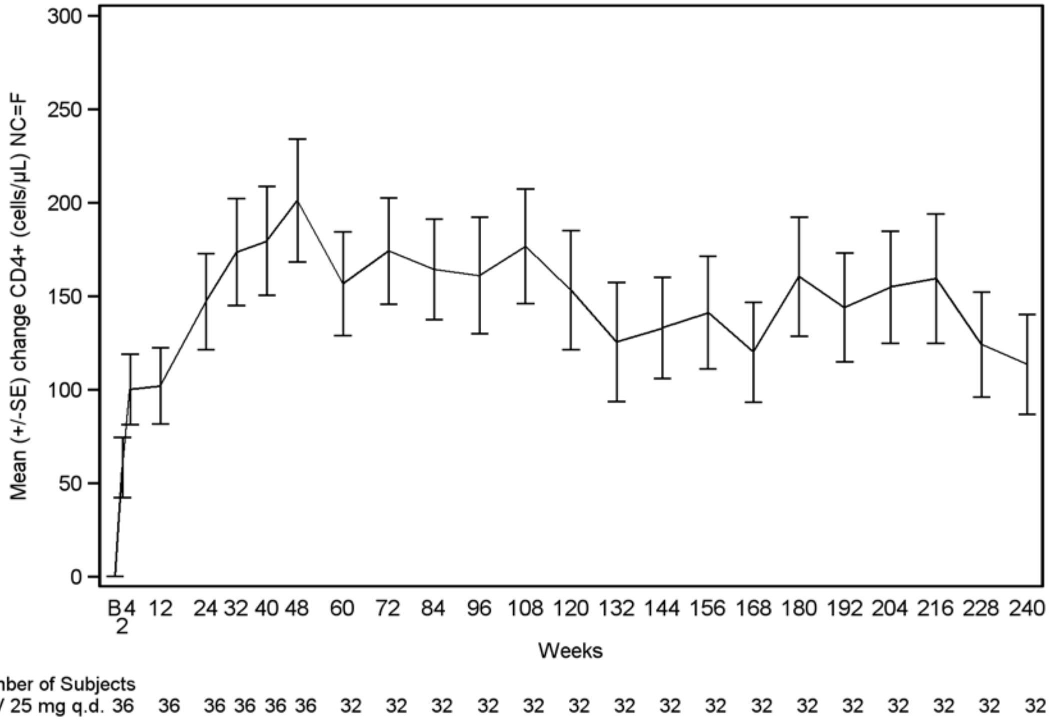

B: baseline; NC=F: non-completer = failure; qd: once-daily; RPV: rilpivirine; SE: standard error

Supplementary Figure 2: Individual viral load profiles over time (Intent-to-treat population)

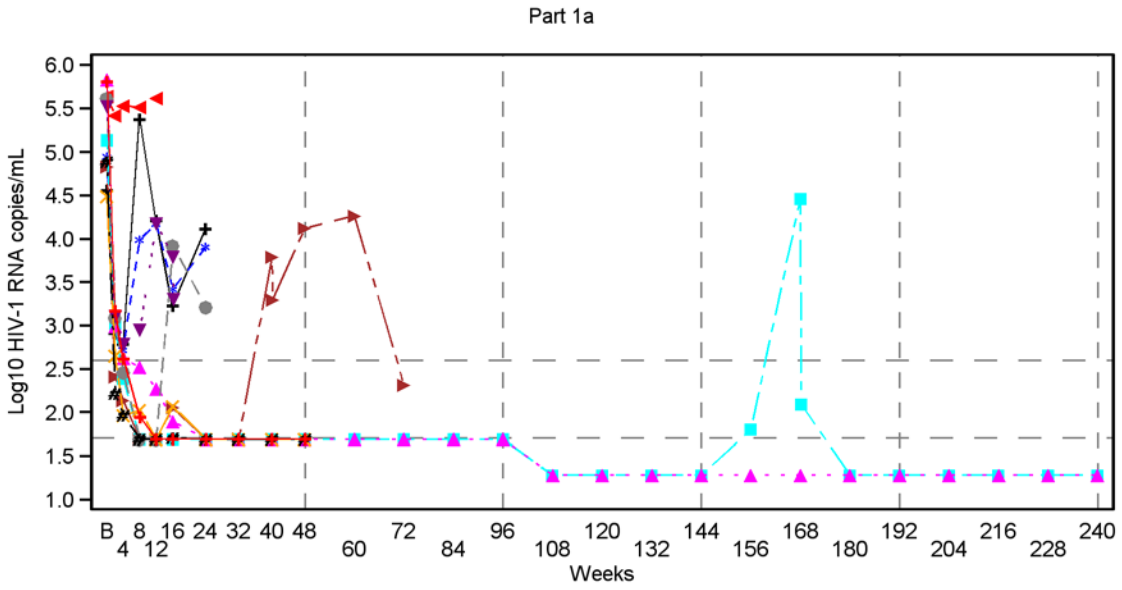

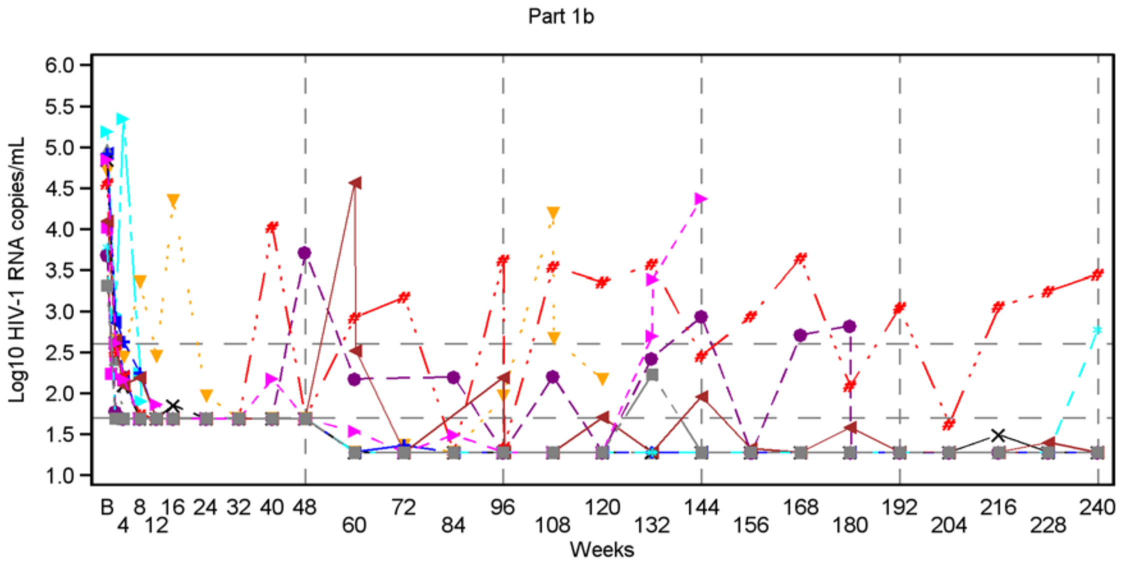

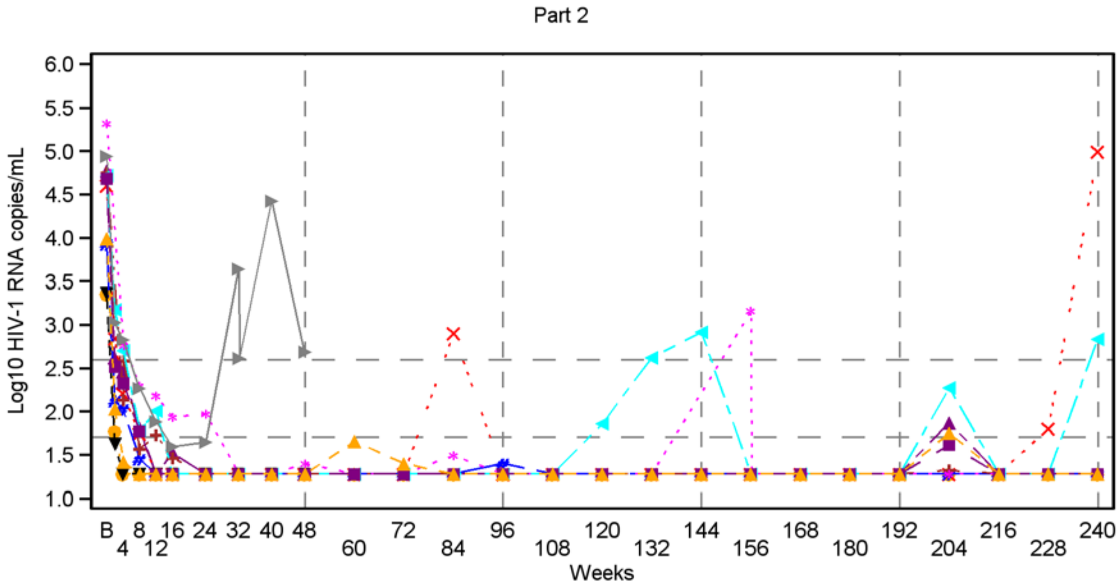

Note: each symbol represents an individual patient
B: baseline; HIV: human immunodeficiency virus; RNA: ribonucleic acid; RPV: rilpivirine;

Supplementary Text

The TLOVR algorithm and FDA snapshot algorithm

TLOVR

U.S. Food and Drug Administration, Center for Drug Evaluation and Research. Guidance for industry: antiretroviral drugs using plasma HIV RNA measurements — clinical considerations for accelerated and traditional approval. Washington, D.C.: Department of Health and Human Services, October 2002. Or describe in full: The TLOVR algorithm for determining response and loss of response (i.e., rebound) requires confirmation at 2 consecutive visits, and participants who drop out of the trial are considered as nonresponders after discontinuation (regardless of the reason). Additionally, intermittent values are imputed according to "time-to" approach, i.e., retain response if loss of response is not yet acquired. Furthermore, any missing viral load results preceding loss of response or discontinuation will be imputed as failures. Participants are considered failures at all visits after confirmed virologic rebound, even if confirmed resuppression (plasma viral load < 50 HIV-1 RNA copies/mL, or < 400 HIV-1 RNA copies/mL) is observed. Participants who switched N(t)RTIs for tolerability reasons will not be counted as failures in the analysis.

FDA Snapshot

U.S. Department of Health and Human Services Food and Drug Administration Center for Drug Evaluation and Research (CDER). Human Immunodeficiency Virus-1 Infection: Developing Antiretroviral Drugs for Treatment Guidance for Industry. November 2015.
